# Early urinary protein changes during tumor formation in a NuTu-19 tail vein injection rat model

**DOI:** 10.1038/s41598-020-68674-z

**Published:** 2020-07-16

**Authors:** Jing Wei, Na Ni, Wenshu Meng, Yuhang Huan, Youhe Gao

**Affiliations:** 1 Department of Biochemistry and Molecular Biology, Beijing Normal University, Gene Engineering Drug and Biotechnology Beijing Key Laboratory, Beijing, 100875 China; 2 Department of Biochemistry and Molecular Biology, College of Basic Medicine, Chongqing Medical University, Chongqing, 400016 China

**Keywords:** Proteomics, Diagnostic markers, Predictive markers, Cancer, Cancer models, Lung cancer, Tumour biomarkers

## Abstract

Early detection of cancer is essential for effective intervention. Urine has been used to reflect early changes in various tumor-bearing models. However, urine has not been used to predict whether tumors will form in animal models. In this study, a cancer model was established by tail vein injection of 2 million NuTu-19 tumor cells. Urine samples were randomly selected from tumor-forming and non-tumor-forming rats on day 0/12/27/39/52 and were analyzed by label-free and parallel reaction monitoring targeted proteomic quantitative analyses. In tumor-forming rats, differential proteins were associated with tumor cell migration, TGF-β signaling and the STAT3 pathway. A total of 9 urinary proteins showed significant changes in the early phase of lung tumor formation in all eight tumor-bearing rats. Differential proteins in non-tumor-forming rats were associated with glutathione biosynthesis, IL-12 signaling and vitamin metabolism. A total of 12 urinary proteins changed significantly in the early phase in all seven non-tumor-forming rats. Our small-scale pilot study indicated that (1) the urinary proteome reflects early changes during lung tumor formation and that (2) the urinary proteome can distinguish early tumor-forming rats from non-tumor-forming rats.

## Introduction

The lungs are common sites for cancer metastasis because of their specific immunologically permissive environment^1^. Therefore, various cancers, such as breast cancer, skin melanoma, colorectal cancer, sarcoma and pancreatic cancer, metastasize more easily to the lungs than to other tissues^2^. Lung metastasis is a lethal determinant in many cancers^3^. The prognosis of patients with malignant lung tumors is very poor, with a low 5-year survival rate, and immunotherapy and chemotherapy have had limited success in reversing lung cancer progression^4,5^. Therefore, there is an urgent need to identify biomarkers for the early detection of lung tumors and even for the prediction of whether tumors will form in the lungs. The discovery of such biomarkers will enable the development of effective therapies for metastatic lung cancer patients.

Urine, as the filtrate of the blood, does not need to remain stable in composition; therefore, it is an ideal biomarker source, accumulating molecules that reflect all changes throughout the body, possibly even early and small pathological changes^6,7^. Urinary proteomics analysis has already been applied for the clinical detection of lung cancer in patients^8,9^. However, the urinary proteome is easily affected by various factors, such as age, sex, diet and medication, especially in the context of clinical urine samples^7^. Experiments using animal models will help to minimize these influential factors and will establish the direct relationships between diseases and corresponding changes in urinary proteins^10^. In addition, the exact starting point and the entire tumor progression period can be controlled in animal model studies, making it possible to collect urine samples in early stages.

In our previous studies, we used urinary proteomes to examine early alterations that occur before pathological changes or clinical manifestations in various tumor-bearing animal models, such as a subcutaneous Walker 256 (W256) rat model^11^, an intracerebral W256 rat model^12^, a glioma rat model^13^, and a W256 lung metastasis rat model^14^. However, there have been no studies on whether urine can be used to predict tumor formation. Therefore, in this experiment, we sought to analyze the differences in urinary proteomes between animals that formed tumors and those that did not.

**Figure 1:**
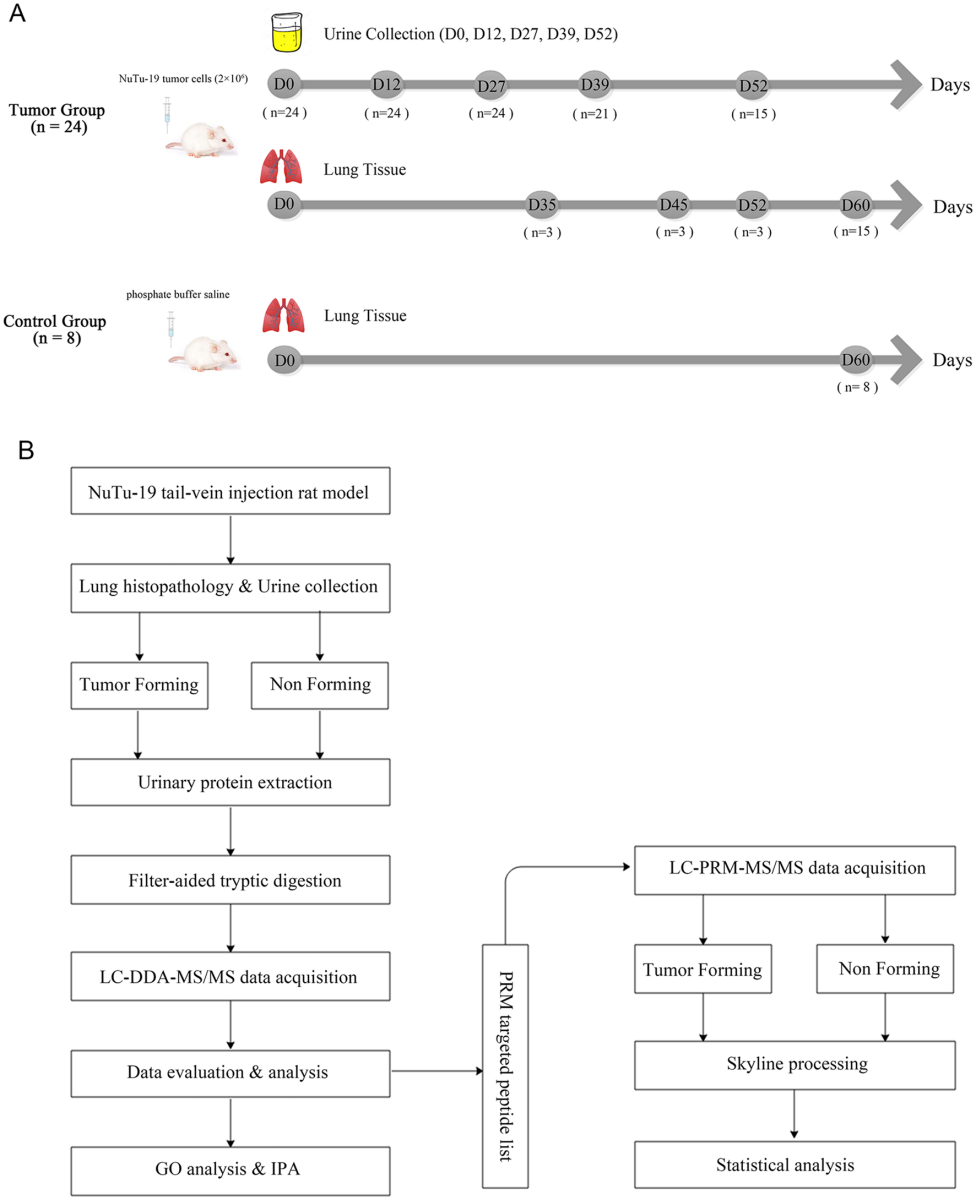
Workflow for the early detection of tumor-forming and non-tumor-forming processes. (**A**) Experimental design of the NuTu-19 tail vein injection rat model. In the tumor group, urine samples were collected on days 0, 12, 27, 39 and 52 after tail vein injection of NuTu-19 cells. Lung tissues were harvested on days 35, 45, 52 and 60. In the control group, all lung tissues were harvested on day 60. (**B**) Urine sample preparation and data analysis for tumor-forming and non-tumor-forming rats. The urinary proteomes of the two groups were identified using liquid chromatography coupled with tandem mass spectrometry (LC–MS/MS) analysis. Then, parallel reaction monitoring (PRM) targeted proteomic quantitative analysis was used to validate candidate biomarkers for the early detection of tumor-forming and non-tumor-forming processes.

Tail vein injection is commonly used to establish metastatic lung carcinoma animal models^15^. In this study, a cancer rat model was established through tail vein injection of two million NuTu-19 tumor cells. Urine samples were collected from the NuTu-19-injected rats on days 0, 12, 27, 39, and 52. Hematoxylin and eosin (HE) staining was used to monitor lung tumor growth. The numbers of tumor-forming and non-tumor-forming rats were recorded after two months. The urinary proteomes were first analyzed with a label-free proteomic method, and candidate biomarkers in tumor-forming and non-tumor-forming rats were then validated by parallel reaction monitoring (PRM) targeted quantification. This study was designed to identify early urinary proteome changes in tumor-forming and non-tumor-forming rats. A technical flowchart is presented in Fig. 1.

## Materials and methods

### Animal model experiment

Female Wistar rats (n = 32, 150 ± 20 g) were purchased from Beijing Vital River Laboratory Animal Technology Co., Ltd. All animals were housed with free access to water and food under standard laboratory conditions. The indoor temperature was 21 ± 2 °C, the humidity was 65–70%, and the cycle conditions were set to 12 h/12 h light/dark. This research was approved by Peking Union Medical College (Approval ID: ACUC-A02-2014-008), with methods and protocols performed in accordance with guidelines for animal research.

The NuTu-19 ovarian cancer cells were purchased from MEIXUAN Biological Science and Technology, Ltd. (Shanghai, China). Before tail vein injection, the NuTu-19 tumor cells were stained with 0.4% trypan blue to estimate their cell viability. Only 95% viable tumor cells were used for the following tail vein injection.

Female Wistar rats (n = 32) were randomly divided into two groups: the NuTu-19 tail vein injection group (n = 24) and the control group (n = 8). The NuTu-19 tail vein injection group was injected with 2 × 10^6^ viable NuTu-19 cells in 100 μL of PBS. The control group was injected with the same volume of PBS via the tail vein^14^.

### Histopathology

For lung tissue histopathology, nine rats in the NuTu-19 tail vein injection group were randomly sacrificed on days 35, 45, and 52 (n = 3 for each time point). Another fifteen NuTu-19 tail vein-injected rats and eight control rats were sacrificed on day 60. Whole lung tissues were fixed in 4% formalin and embedded in paraffin. Then, paraffin sections (4 μm thick) were stained with HE to reveal the metastatic tumors^14^.

### Urinary protein extraction

Urine samples were collected from the NuTu-19 tail vein injection group on days 0, 12, 27, 39, and 52. Each rat was placed in a metabolic cage for 12 h without food and water for collection of urine (at least 8 mL). Then, urine samples were centrifuged at 3,000 × *g* for 30 min at 4 °C before storage at − 80 °C.

For urinary protein extraction, urine samples were first centrifuged at 12,000 × *g* for 30 min at 4 °C. Then, three times the volume of ethanol was added for precipitating proteins at − 20 °C overnight. Lysis buffer (8 mol/L urea, 2 mol/L thiourea, 50 mmol/L Tris, and 25 mmol/L DTT) was added to re-dissolve the pellets. Finally, the supernatants were quantified using a Bradford assay.

### Tryptic digestion

The proteins were digested with trypsin (Trypsin Gold, Mass Spec Grade, Promega, Fitchburg, Wisconsin, USA) using filter-aided sample preparation methods^16^. Briefly, aliquots of 100 μg of protein were loaded onto 10-kDa-cutoff filter devices (Pall, Port Washington, NY) and washed twice with UA (8 M urea in 0.1 M Tris–HCl, pH 8.5) and 25 mmol/L NH_4_HCO_3_ at 14,000 × *g* and 18 °C for 40 min. Then, each urinary protein was denatured with 20 mM DTT at 37 °C for 1 h and alkylated with 50 mM iodoacetamide (IAA) for 40 min in the dark. After being washed twice with UA and 25 mmol/L NH_4_HCO_3_, the denatured proteins were resuspended in 25 mmol/L NH_4_HCO_3_ and digested with trypsin (enzyme:protein ratio of 1:50) at 37 °C for 14 h. The collected peptides were desalted using Oasis HLB cartridges (Waters, Milford, MA) and then dried by vacuum evaporation (Thermo Fisher Scientific, Waltham, MA)^14^.

### LC–MS/MS analysis

Twenty urine samples from four randomly selected NuTu-19 lung tumor-forming rats and another 20 urine samples from four randomly selected non-tumor-forming rats were chosen for MS analysis. For analysis, 1 µg of peptide from each sample was loaded into a trap column (75 µm × 2 cm, 3 µm, C18, 100 Å) at a flow rate of 0.25 μL/min and separated with a reversed-phase analytical column (75 µm × 250 mm, 2 µm, C18, 100 Å). The peptides were eluted with a gradient extending from 5 to 30% buffer B (0.1% formic acid in 80% acetonitrile) for 60 min and were then analyzed with an Orbitrap Fusion Lumos Tribrid Mass Spectrometer (Thermo Fisher Scientific)^17^. To obtain MS data, survey MS scans were acquired in the Orbitrap using a range of 350–1,550 m/z with the resolution set to 120,000. MS/MS scans for each full scan in top-speed mode (3 s) were selected for collision-induced dissociation fragmentation with a resolution of 30,000 in the Orbitrap, and dynamic exclusion was employed with a 30-s window to prevent repeated selection of the same peptide. The normalized collision energy for the HCD-MS2 experiments was set to 30%, the charge-state screening was set to + 2 to + 7, and the maximum injection time was 45 ms. Each sample was analyzed with two technical replicates.

### Label-free quantification

The label-free raw MS/MS data files were searched using Mascot software (version 2.5.1, Matrix Science, London, UK) against the SwissProt rat database (released in February 2017; contains 7,992 sequences). The search parameters included a parent ion tolerance of 10 ppm and a fragment ion mass tolerance of 0.02 Da. Carbamidomethylation of cysteine was set as a fixed modification, and oxidation of methionine was considered a variable modification. The specificity of trypsin digestion was set for cleavage after K or R, and two missed trypsin cleavage sites were allowed.

The raw MS data files for NuTu-19 tumor-forming rats (n = 40) and non-tumor-forming rats (n = 40) were processed using Progenesis software (version 4.1, Nonlinear, Newcastle upon Tyne, UK) for label-free quantification^18^. Features with only one charge or with more than five charges were excluded from the analyses. Protein abundance was calculated from the sum of all unique peptide ion abundances for a specific protein in each run and normalized by the median abundance of the commonly identified proteins. The normalization of abundances was required by the software to allow comparisons to be made across different sample runs. For further quantification, all peptides (with Mascot scores > 30 and *P* values < 0.01) of an identified protein were included. Proteins identified by at least one peptide were retained.

### Parallel reaction monitoring (PRM) analysis

Twenty urine samples from the remaining four NuTu-19 tumor-forming rats and fifteen urine samples from the remaining three non-tumor-forming rats were used for PRM verification analysis. The peptides were first pooled (2 μg of each sample) for LC-DDA-MS/MS analysis to build a spectrum library with 6 runs for definition of the targeted peptide retention times. Then, individual LC-PRM-MS/MS analyses were performed. For analysis, 900 ng of pooled peptides was loaded into a trap column (75 µm × 2 cm, 3 µm, C18, 100 Å) at a flow rate of 0.25 μL/min and separated with a reversed-phase analytical column (75 µm × 250 mm, 2 µm, C18, 100 Å). The peptides were eluted with a gradient extending from 5 to 30% buffer B (0.1% formic acid in 80% acetonitrile) and were then analyzed with an Orbitrap Fusion Lumos Tribrid Mass Spectrometer (Thermo Fisher Scientific, Waltham, MA)^17^. The elution time was set to 120 min. The MS data were acquired using the following steps: full scans (m/z 350–1,550) were acquired with a resolution of 60,000, PRM scans (m/z 200–2000) were run at a resolution of 30,000, the retention time window was set to ± 2 min, and targeted peptides were isolated using a 1.6-m/z window. The 30% HCD of the normalized collision energy was used, and the maximum injection time was 60 ms.

The raw pooled MS/MS data files were searched using Thermo Proteome Discover 2.1.0.81 against the SwissProt rat database (released in February 2017; contains 7,992 sequences) with precursor and fragment mass tolerances of 10 ppm and 0.02 Da, respectively. The other parameters were set as follows: trypsin digestion, two maximum missed cleavage sites, oxidation (+ 15.995 Da) of methionine as a dynamic modification, and carbamidomethylation (+ 57.021 Da) of cysteine as a static modification^19^. The protein and peptide FDRs were set to 1%. Skyline software (version 3.6.1) was used to build a spectrum library and filter the targeted peptides for PRM analysis^20^. Two to six peptides of each targeted protein were selected using the following criteria: (1) identified in untargeted analysis with a Mascot score > 30 and a *P* < 0.01, (2) digested by trypsin [KR/P] with a maximum of 2 missed cleavages, (3) 8–35 amino acid residues in length, (4) exclusion of the first 25 N-terminal amino acids, and (5) structurally modified by carbamidomethyl (C) and oxidation (M). Only the unique peptides of each protein were used for the subsequent targeted quantification. In total, 34 targeted proteins with 107 peptides were selected in the tumor-forming group, while 23 targeted proteins with 130 peptides were selected in the non-tumor-forming group. The retention time (RT) segment was set to 4 min for each targeted peptide, with the expected RT in the center based on the pooled sample analysis. Ultimately, for the tumor-forming group, 29 proteins with 80 peptides were selected for further PRM validation (Table S1A). A technical reproducibility assessment of the PRM assay indicated that there were 71 targeted peptides with CV values less than 20% (Figure S1A). For the non-tumor-forming group, 23 urinary proteins with 115 peptides were selected for PRM validation (Table S1B). Among these targeted peptides, 113 had CV values less than 20% (Figure S1B).

Individual peptide samples (900 ng of each sample) were then analyzed by PRM assays. The transition settings in Skyline were as follows: the precursor charges were set to + 2, + 3, and + 4; the ion charges were set to + 1 and + 2; the ion types were set to y, b, and p; the product ion selection was set from ion 3 to the last ion; the ion match tolerance was set to 0.02 m/z; the number of product ions was set to 6; “auto-select all matching transitions” was activated; and the minimum dotp was set to 0.7. The details of the transition are listed in Table S2. Each protein was quantified using the summation of the fragment area from its corresponding transitions. Prior to statistical analysis, summation of the fragment area was performed by log2 transformation. The differential proteins were identified using one-way ANOVA, and the significance threshold was set at a *P* value < 0.05.

### Functional enrichment analysis

All differential urinary proteins in the tumor-forming and non-tumor-forming groups on days 12, 27, 39 and 52 were assigned gene symbols using the Database for Annotation, Visualization and Discovery (DAVID)^21^ and analyzed with the Gene Ontology (GO) database based on the biological process category. The biological pathways and diseases/functions related to the differential proteins were analyzed at four time points with Ingenuity Pathway Analysis (IPA) software (Ingenuity Systems, Mountain View, CA, USA).

### Statistical analysis

The average normalized abundance for each sample was used for statistical analysis. The levels of the proteins identified in the tumor-forming and non-tumor-forming groups on days 12, 27, 39 and 52 were compared with the levels in those groups on day 0. The differential proteins were selected with the following criteria: number of unique peptides ≥ 2; fold change ≥ 1.5 or ≤ 0.67; *P* < 0.05 in a two-sided unpaired *t* test; and a greater normalized abundance in every rat in the high-abundance group than in the rats in the low-abundance group. The *P* values for the group differences were adjusted by the Benjamini & Hochberg method^22^. Group differences resulting in adjusted *P* values < 0.05 were considered statistically significant. All results are expressed as the means ± standard deviations.

## Results

### Characterization of the NuTu-19 tail vein-injected rats

**Figure 2:**
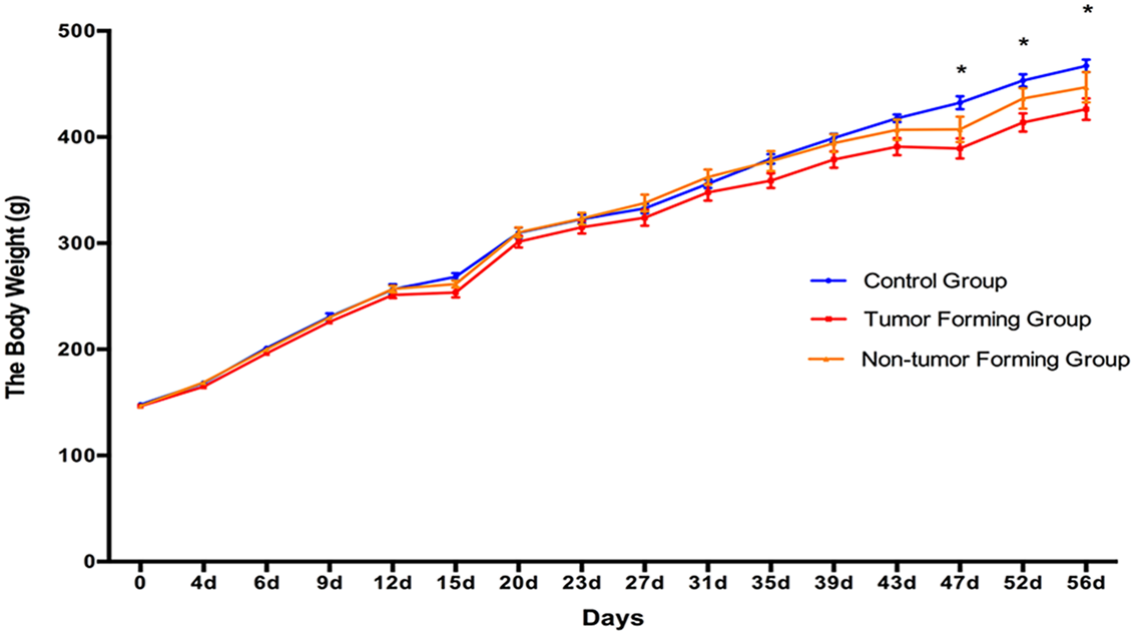
Body weight changes in NuTu-19 tail vein-injected rats. The results are shown as the means ± SD for the tumor-forming group (n = 8), the non-tumor-forming group (n = 7), and the control group (n = 8) (*P < 0.05).

**Figure 3:**
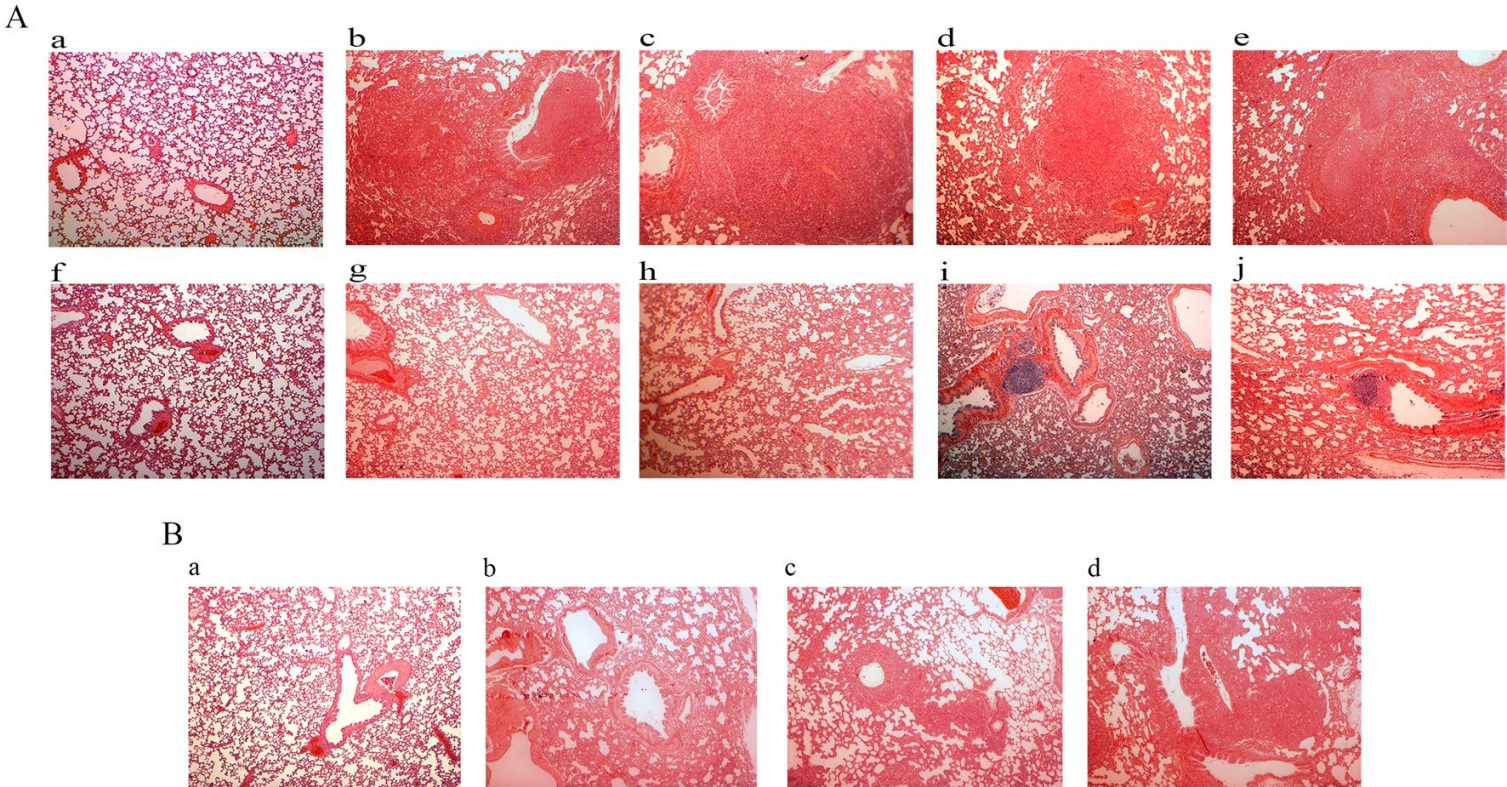
Pathological changes in lung tissues. (**A**) Pathological changes in NuTu-19-injected tumor-forming and non-tumor-forming rats on day 60. (**a**, **f**) Control group injected with PBS via the tail vein. (**b**–**e**) Four NuTu-19-injected tumor-forming rats used for the discovery phase on day 60. (**g**–**j**) Four NuTu-19-injected non-tumor-forming rats on day 60. (**B**) Changes in NuTu-19 tail vein-injected rats at different time points. (**a**) Control group injected with PBS via the tail vein. (**b**–**d**) NuTu-19 tail vein injection rats on days 35, 45, and 52. The magnification was 40 × for the images of HE staining.

Thirty-two female Wistar rats (150 ± 20 g) were divided into the control group (n = 8) and the NuTu-19 tail vein injection group (n = 24). The rats in the control group had normal daily activity with shiny hair, while some of the NuTu-19 tail vein-injected rats showed piloerection in the late stages. Fifteen rats were sacrificed on day 60. Eight rats showed obvious lung tumors, while the other seven rats did not. Representative images of the lung histopathology are presented in Fig. 3. The average body weight of the tumor-forming rats was significantly lower than that of the control rats until day 47 (*P* < 0.05); in addition, the average body weight of the non-tumor-forming group was lower than that of the control group, but the difference was not significant (Fig. 2).

The lung pathological changes in the tumor-forming rats and the non-tumor-forming rats on day 60 are shown in Fig. 3A. On day 60, all tumor-forming rats showed severe lung metastatic nodules scattered throughout the lung parenchyma. The lung tissue and the alveolar structures showed severe destruction. The non-tumor-forming rats did not show metastatic tumor nodules on day 60 but had large numbers of lymphocytes surrounding the bronchi. The pathological changes in the NuTu-19 tail vein-injected rats at different time points are shown in Fig. 3B. The lung metastatic tumor nodules became apparent 45 days after tail vein injection of NuTu-19 cells, indicating that days 12 and 27 fell within the early stages of lung tumor formation.

### Urinary proteome changes

Twenty urine samples from 4 tumor-forming and 4 non-tumor-forming rats at five time points (days 0, 12, 27, 39, and 52) were subjected to label-free quantification, with two technical replicates per sample. In total, 532 proteins with at least two peptides were identified in tumor-forming rats (Table S3A). In non-tumor-forming rats, a total of 517 urinary proteins with at least two peptides were identified (Table S3B). Overall, 180 and 327 significantly changed proteins were identified in the tumor-forming and non-tumor-forming rats, respectively, according to the screening criteria. Specifically, on days 12, 27, 39, and 52, the tumor-forming rats exhibited 99, 120, 123, and 143 differential proteins, respectively, while the non-tumor-forming rats exhibited 49, 179, 149 and 262 differential proteins, respectively. The details of these differential proteins at different time points are presented in Tables S4 and S5. The overlapping differential proteins between tumor-forming and non-tumor-forming rats are presented in Figure S2.

### Functional comparison analysis

**Figure 4:**
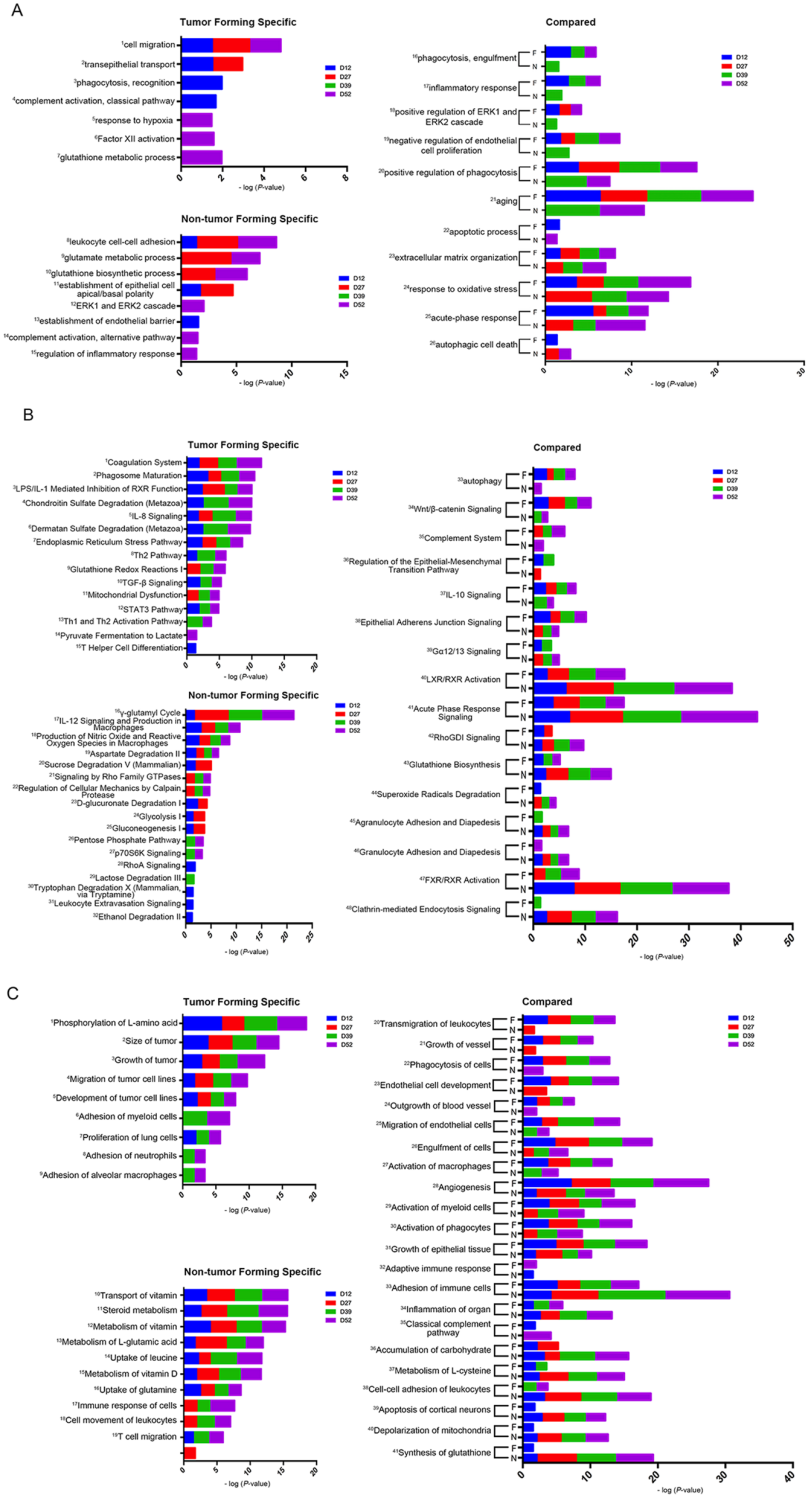
Functional enrichment analysis of tumor-forming and non-tumor-forming rats. Dynamic changes in biological processes (**A**), pathways (**B**) and disease and functional terms (**C**) at multiple time points were classified. Each part is divided into tumor-specific, non-tumor-specific and compared analyses. All enriched terms had *P* values < 0.05. F represents the tumor-forming group, and N represents the non-tumor-forming group.

To determine why some rats formed lung tumors while others had not by day 60, functional annotation was performed for the differential proteins in tumor-forming and non-tumor-forming rats at four time points (days 12, 27, 39, and 52) using both the DAVID and IPA. The biological processes, pathways and disease/functions were compared between the tumor-forming and non-tumor-forming rats. Representative enriched functional terms are presented in Fig. 4. All these representative terms were identified with a significance threshold of *P* < 0.05.

Upon comparing the functional enrichment analysis results between the tumor-forming and non-tumor-forming rats, we found that some of the functional terms were enriched only in tumor-forming rats, such as the cell migration and transepithelial transport terms (Fig. 4A1 and 2, respectively); the coagulation system, IL-8 signaling, and TGF-β signaling terms (Fig. 4B1, 5, and 10, respectively); and the phosphorylation of L-amino acid, size of tumor, growth of tumor, and migration of tumor cell line terms (Fig. 4C1, 2, 3, and 4, respectively). Conversely, some functional terms were enriched only in non-tumor-forming rats, including the leukocyte cell–cell adhesion, glutamate metabolic process, and glutathione biosynthetic process terms (Fig. 4A8, 9, and 10, respectively); the γ-glutamyl cycle, IL-12 signaling and production in macrophages, and production of nitric oxide and reactive oxygen species in macrophages terms (Fig. 4B16, 17, and 18, respectively); and the transport of vitamin, steroid metabolism, metabolism of vitamin, and metabolism of L-glutamic acid terms (Fig. 4C10, 11, 12, and 13, respectively).

Some of the functional terms were enriched in both tumor-forming and non-tumor-forming rats but exhibited stronger tendencies for enrichment in tumor-forming rats. Such terms included the phagocytosis engulfment and positive regulation of phagocytosis terms (Fig. 4A16 and 20, respectively); the phagocytosis of cells, engulfment of cells and activation of phagocytes terms (Fig. 4C22, 26, and 30, respectively); the positive regulation of ERK1 and ERK2 cascade term (Fig. 4A18); the autophagy, Wnt/β-catenin signaling, complement system, and IL-10 signaling terms (Fig. 4B33, 34, 35, and 37, respectively); and the growth of vessel, endothelial cell development, outgrowth of blood vessel, angiogenesis, and growth of epithelial tissue terms (Fig. 4C21, 23, 24, 28, and 31, respectively). All these terms were more associated with cancer metastatic progression than with other processes. In addition, some of the commonly enriched terms were enriched in both groups of rats but were more prominent in non-tumor-forming rats. Such terms included the glutathione biosynthesis and superoxide radical degradation (Fig. 4B43 and 44, respectively); the accumulation of carbohydrate, metabolism of L-cysteine, depolarization of mitochondria, and synthesis of glutathione terms (Fig. 4C36, 37, 40, and 41, respectively); the agranulocyte adhesion and diapedesis term (Fig. 4B45); the cell–cell adhesion of leukocytes term (Fig. 4C38); the RhoGDI signaling and clathrin-mediated endocytosis signaling terms (Fig. 4B42 and 48, respectively); and the adhesion of immune cells and organ inflammation terms (Fig. 4C33 and 34, respectively).

Some additional enriched functional terms also deserve attention. For example, the apoptotic process (Fig. 4A22) and classical complement pathway (Fig. 4C35) terms were overrepresented in tumor-forming rats only on day 12 and were overrepresented in non-tumor-forming rats only on day 52. In contrast, the adaptive immune response term (Fig. 4C32) was overrepresented in tumor-forming rats only on day 52 and was overrepresented in non-tumor-forming rats only on day 12. These different functional terms that were enriched in tumor-forming and non-tumor-forming rats will help us to better understand lung tumor formation associated with lung cancer metastasis.

### Parallel reaction monitoring (PRM) validation

Comparison of the differential urinary proteins in tumor-forming and non-tumor-forming rats revealed 125 common proteins between the groups (Figure S2). These 125 common proteins were excluded from the subsequent PRM analysis. Some early differential proteins (days 12 and 27) identified in tumor-forming and non-tumor-forming rats were validated by PRM. The details are presented in Table S6.

**Figure 5:**
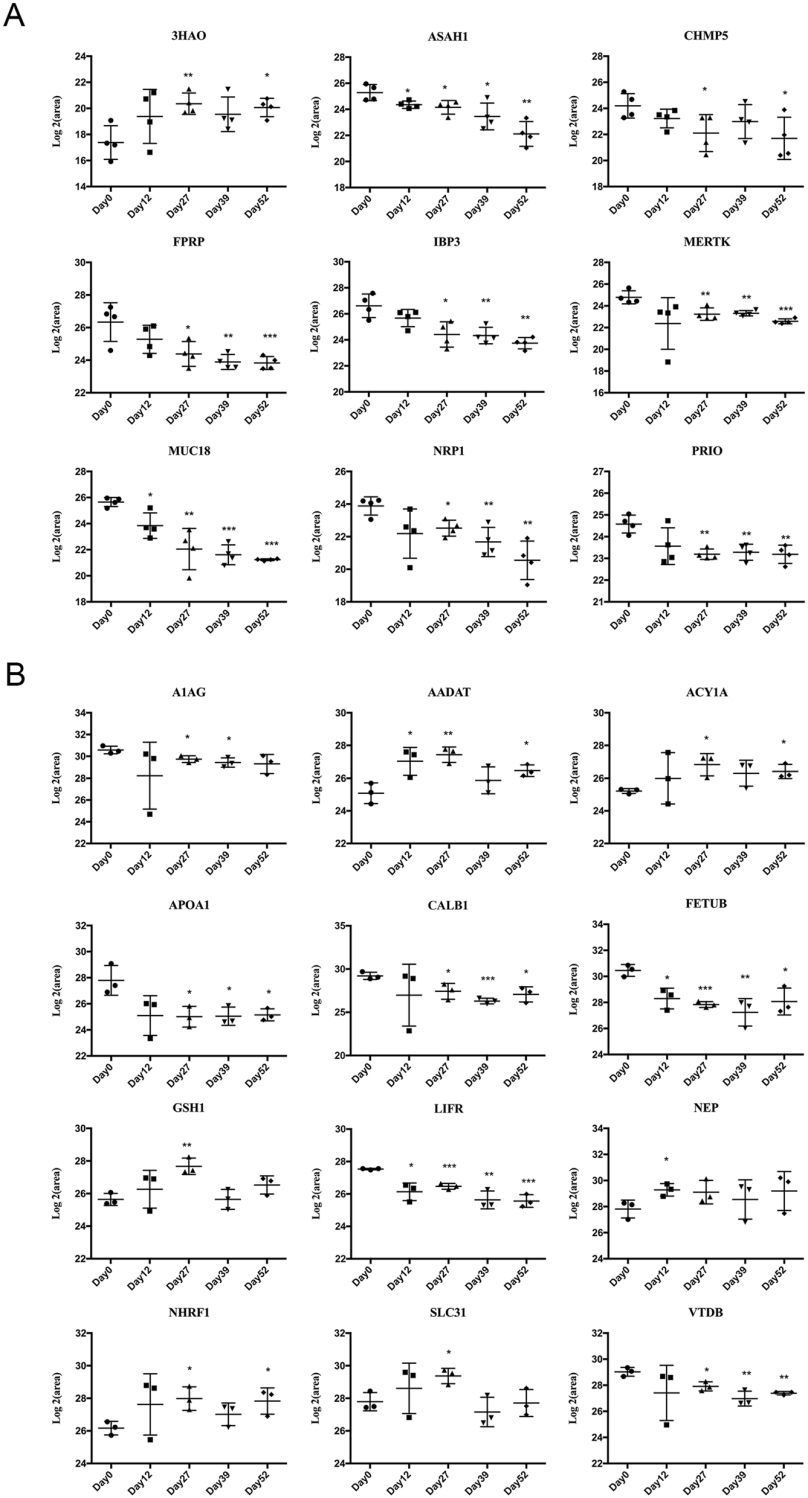
Validation of the expression levels of candidate urinary biomarkers from NuTu-19-injected tumor-forming and non-tumor-forming rats by PRM quantification. (**A**) Tumor-forming rats. (**B**) Non-tumor-forming rats. The x-axis shows the different stages after injection of NuTu-19 tumor cells via the tail vein. The y-axis shows the log2 area of the intensity based on PRM quantification (*P < 0.05; **P < 0.01; ***P < 0.001).

With regard to the tumor-forming group, proteins were subjected to PRM validation in the remaining four tumor-forming rats. A total of 23 proteins were successfully quantified, and 14 of them were significant. The following criteria were used for further selection: (1) the protein had the same fold change trend from the day 0 value as that revealed by label-free quantification, (2) the protein was identified on day 12 or day 27, and (3) the protein had a human ortholog. Ultimately, 9 urinary proteins (3HAO, ASAH1, CHMP5, FPRP, IBP3, MERTK, MUC18, NRP1, and PRIO) changed significantly during the early phase of lung tumor formation (Fig. 5A). The details are listed in Table 1.

**Table 1:** Candidate biomarkers for the early detection of lung tumor formation.

UniProt ID	Human ortholog	Protein name	Trend	Lung cancer biomarkers	Lung cancer pathology/mechanism	Ovarian cancer metastasis
P15473	P17936	Insulin-like growth factor-binding protein 3 (IBP3)	↓	S^35^; S, T^36^; B, S^37^	^38,51^	^39^
Q62786	Q9P2B2	Prostaglandin F2 receptor negative regulator (FPRP)	↓	–	–	–
Q9EPF2	P43121	Cell surface glycoprotein MUC18 (MUC18)	↓	–	–	–
Q9QWJ9	O14786	Neuropilin-1 (NRP1)	↓	T^40^	^41,52^	^42^
P13852	P04156	Major prion protein (PRIO)	↓	–	–	–
P57097	Q12866	Tyrosine-protein kinase Mer (MERTK)	↓	–	^43^	–
Q4QQV8	Q9NZZ3	Charged multivesicular body protein 5 (CHMP5)	↓	–	–	–
Q6P7S1	Q13510	Acid ceramidase (ASAH1)	↓	–	–	^53^
P46953	P46952	3-Hydroxyanthranilate 3,4-dioxygenase (3HAO)	↑	–	–	–

*S* serum, *T* tissue, *B* bronchoalveolar lavage fluid.

With regard to the non-tumor-forming group, 23 proteins were subjected to PRM validation in the remaining three non-tumor-forming rats. All 23 of these proteins were successfully quantified, and 15 of them were significant. According to the previously described criteria, a total of 12 differential proteins (A1AG, AADAT, ACY1A, APOA1, CALB1, FETUB, GSH1, LIFR, NEP, NHRF1, SLC31, and VTDB) were ultimately identified in the early phase of the non-tumor-forming process in the lung (Fig. 5B).

## Discussion

The early detection of tumors is essential for effective intervention. Although intraperitoneal dissemination is considered the most common mechanism of spread, ovarian cancer can also metastasize through blood vessels to the parenchyma of the lungs^23^. Urinary proteomes have already been used to reflect early changes in different types of cancer animal models^8,11,13^; however, urine has not been used to predict whether tumors will form in animal models. In this study, a cancer model was established by tail vein injection of 2 million NuTu-19 tumor cells. Approximately half of the fifteen injected rats formed metastatic lung carcinoma tumors after 60 days. We therefore sought to detect the different urinary proteome changes in tumor-forming and non-tumor-forming rats. After label-free quantification and PRM validation, we ultimately identified 9 differential urinary proteins in the 8 tumor-forming rats and 12 differential urinary proteins in the 7 non-tumor-forming rats during the early phase of lung metastasis.

Some differential proteins in the tumor-forming rats revealed the occurrence of events associated with cancer metastasis cascades and tumor progression. For example, proteins related to the cell migration (Fig. 4A1) and migration of tumor cell lines (Fig. 4C4) terms, which are associated with cancer metastasis cascades^24^, were enriched only in the tumor-forming rats. When cancer cells enter the bloodstream, their interactions with other cell types can help them avoid immune attack and facilitate their passage to and extravasation at distant sites^25^. With regard to the enriched terms, the coagulation system (Fig. 4B1) has been reported to provide an additional layer of immune evasion, which contributes to tumor cell metastasis^24–26^. In addition, TGF-β signaling (Fig. 4B10) has been reported to derive from platelets, and adhered platelets can prevent tumor cell recognition and lysis by NK cells^27^. Furthermore, adhesion of neutrophils (Fig. 4C8) enables tumor cells to travel with these cells in the circulation, which allows the tumor cells to survive intraluminally, adhere to endothelial cells, and be extravasated^28^. Transepithelial transport (Fig. 4A2) and the adhesion of myeloid cells and alveolar macrophages (Fig. 4C6 and 9) have been reported to be associated with the extravasation process^29^. Other enriched functional terms were also associated with tumor progression. For example, tumor size, tumor growth, and tumor cell line development (Fig. 4C2, 3, and 5, respectively) are associated with progression, as are the complement activation classical pathway (Fig. 4A4), IL-8 signaling, the Th2 pathway, the STAT3 pathway, the Th1 and Th2 activation pathways and T helper cell differentiation (Fig. 4B5, 8, 12, 13, and 15, respectively)^30–34^.

Notably, some of the nine differential proteins (3HAO, ASAH1, CHMP5, FPRP, IBP3, MERTK, MUC18, NRP1, and PRIO) in tumor-forming rats have already been reported to be associated with lung cancer biomarkers/pathology/mechanisms or ovarian cancer metastasis. First, insulin-like growth factor-binding protein 3 (IBP3) has been reported to be a lung cancer biomarker in serum, bronchoalveolar lavage fluid and lung tissue^35–37^ and has also been reported to modulate lung tumorigenesis^38^. In the context of epithelial ovarian cancer, IGFBP-3 has been reported to play an important role as an invasion/metastasis suppressor^39^. Second, Neuropilin-1 (NRP1) has been reported to be highly expressed in non-small-cell lung cancer cells (NSCLCs) and to play a key role in the occurrence, development and metastasis of NSCLC^40^. Coexpression of the NRP1 and NRP2 genes has been reported to be significantly correlated with tumor progression through neovascularization in NSCLC^41^. In addition, NRP1 has been reported to be a valuable prognostic marker and a potential molecular therapeutic target for ovarian cancer patients^42^. Third, Mer tyrosine kinase (MERTK) has been reported to be overexpressed in human NSCLC^43^. We suggest that these nine differential proteins may provide a potential panel for the early detection of lung tumor formation.

According to previous research, only a few metastases may ultimately be detectable in end-point assays even when millions of cancer cells are injected into an experimental mouse, indicating that metastasis is an inefficient process^44^. We found that some functional terms were highly enriched specifically in non-tumor-forming rats. For example, (1) the glutamate metabolic process and glutathione biosynthetic process terms (Fig. 4A9 and 10, respectively); the γ-glutamyl cycle and aspartate degradation II terms (Fig. 4B16 and 19, respectively); and the metabolism of l-glutamic acid, uptake of leucine, and uptake of glutamine terms (Fig. 4C13, 14, and 16, respectively) were associated with cancer glutamine metabolism. It has been reported that restricting glutamine metabolism effectively inhibits tumor growth by inducing apoptosis, growth arrest and/or autophagy^45^. Glutamine is first converted into glutamate via glutaminase (GLS/GLS2), and glutamate contributes to the synthesis of glutathione^46^. (2) IL-12 signaling and production in macrophages (Fig. 4B17) has been reported to have extraordinary antitumor efficacy in diverse types of animal cancer models as well as in human cancer clinical trials^47^. (3) The production of nitric oxide and reactive oxygen species in macrophages (Fig. 4B18) has been reported to be due to proinflammatory mediators that participate in the innate immune response^48,49^. (4) Vitamin D transport, steroid metabolism, vitamin D metabolism, and steroid metabolism (Fig. 4C10, 11, 12, and 15, respectively) have been reported to be helpful in the treatment of cancer, especially when vitamin D metabolites or analogs are added to existing therapies^50^. Therefore, we hypothesize that these described processes may contribute to a lack of tumor formation.

We also compared the differential proteins identified in tumor-forming rats and W256 lung metastasis rats^14^ and found that the urinary proteomes of different tumor cell types may be different even when the cells grow in the same organs (Figure S3). Notably, our research ended on day 60; it is unclear whether the non-tumor-forming rats would have formed lung tumors at later stages. Overall, this study was a preliminary study with small numbers of tumor-forming and non-tumor-forming rats. Our results reveal that the urinary proteome can potentially be used to distinguish between tumor-forming and non-tumor-forming lung statuses at an early stage and provide potential clues for early clinical intervention. However, urinary protein biomarkers require further evaluation in large numbers of clinical samples to test their sensitivity and specificity. Some significant pathways identified in non-tumor-forming rats in this study may also have applications for monitoring in cancer treatment and prevention studies.

## Supplementary information

Supplementary file1 (PDF 956 kb)

Supplementary file2 (XLSX 17 kb)

Supplementary file3 (XLSX 68 kb)

Supplementary file4 (XLSX 1197 kb)

Supplementary file5 (XLSX 125 kb)

Supplementary file6 (XLSX 140 kb)

Supplementary file7 (XLSX 16 kb)

## Data Availability

All raw files were uploaded to an online repository database named iProX (www.iprox.org). (URL: https://www.iprox.org/page/PSV023.html;?url=1593789469531ZjU4; Password:9bXh).
