# Supplementary material for: Early urinary protein changes during tumor formation in a NuTu-19 tail vein injection rat model
Source: Sci Rep. 2020 Jul 16;10:11709. doi: 10.1038/s41598-020-68674-z (PMC7367258; doi:10.1038/s41598-020-68674-z)
Supplement: Supplementary file 1 — Supplementary file1 (PDF 956 kb) [file 41598_2020_68674_MOESM1_ESM.pdf]

Supplementary Information

Early urinary protein changes during tumor formation in a NuTu-19 tail vein injection rat model

Jing Wei[1], Na Ni[2], Wenshu Meng[1], Yuhang Huan[1], Youhe Gao[1]*
[1]Department of Biochemistry and Molecular Biology, Beijing Normal University, Gene Engineering Drug and Biotechnology Beijing Key Laboratory, Beijing, 100875, China
[2]Department of Biochemistry and Molecular Biology, College of Basic Medicine, Chongqing Medical University, Chongqing, 400016, China
Corresponding author:
Youhe Gao

Email address: gaoyouhe@bnu.edu.cn

Tel.: +86 10 58804382

Supplementary Figure S1. (A) CV values of 80 PRM targeted peptides for validation of tumor formation. (B) CV values of 115 PRM targeted peptides for validation of a lack of tumor formation.

A

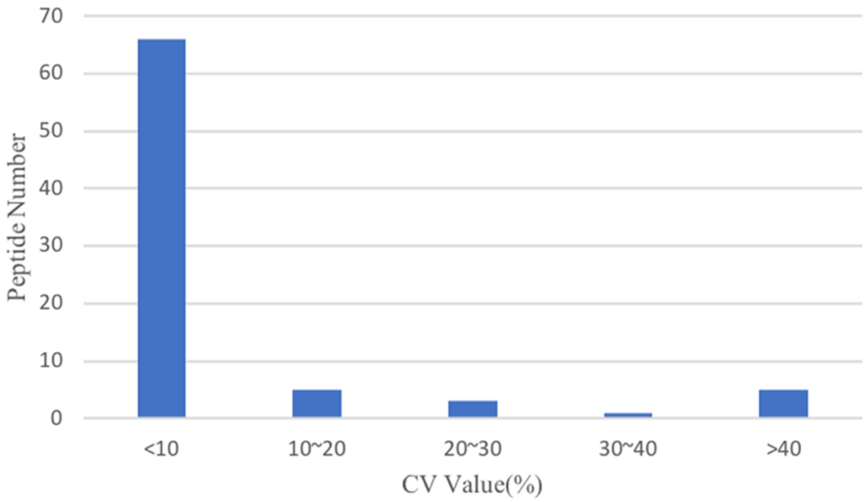

B

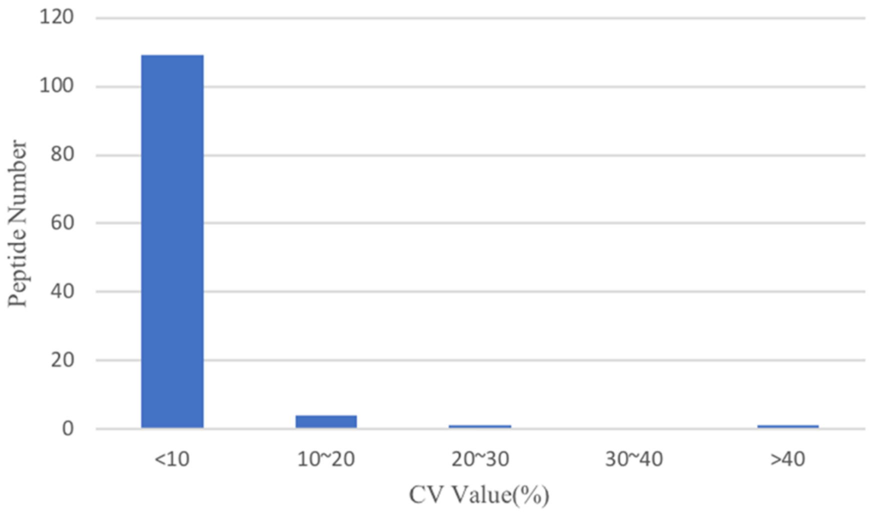

Supplementary Figure S2. Comparison of the differential urinary proteins between the tumor-forming group and the non-tumor-forming group.

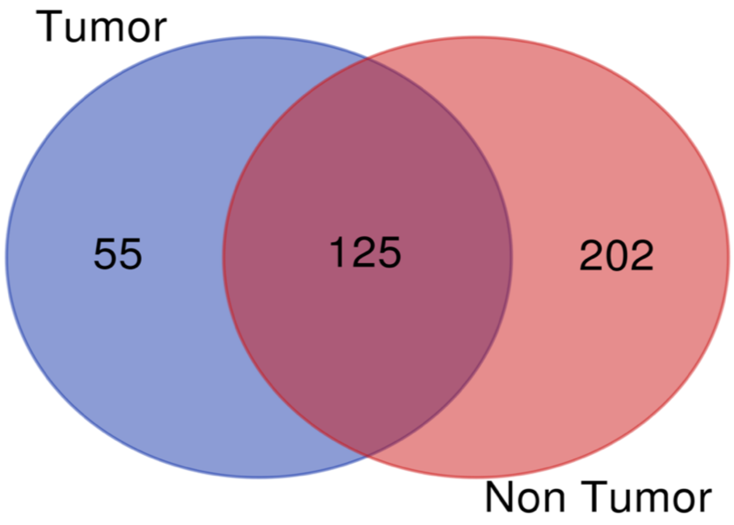

Supplementary Figure S3. Comparison of the differential urinary proteins between Walker 256 tail vein-injected rats and NuTu-19 tail vein-injected rats.

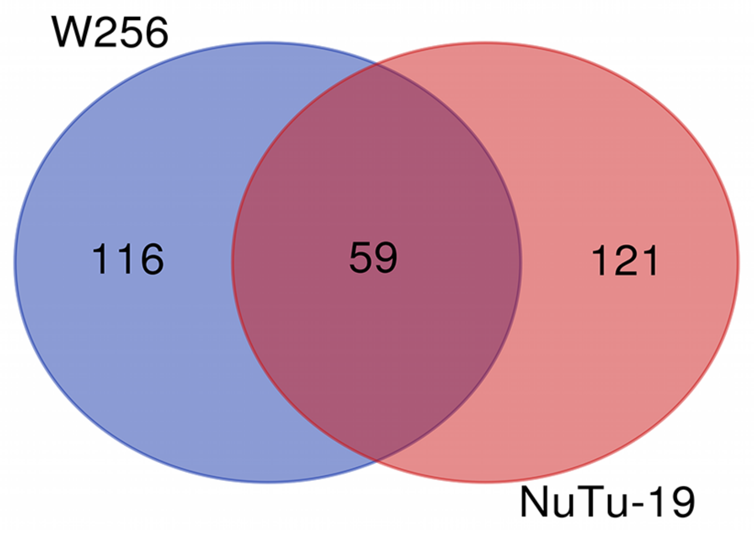

Supplementary Table S1. (A) Retention times of 29 differential proteins with 80 peptides used for tumor-forming PRM validation. (B) Retention times of 23 differential proteins with 115 peptides used for non-tumor-forming PRM validation.

Supplementary Table S2. Transition list used for PRM targeted proteomic quantification in the tumor-forming group (A) and the non-tumor-forming group (B).

Supplementary Table S3. Identification and quantification details of the urinary proteomes identified in the tumor-forming group (A) and the non-tumor-forming group (B).

Supplementary Table S4. Differential proteins identified on days 12, 27, 39, and 52 in the tumor-forming group.

Supplementary Table S5. Differential proteins identified on days 12, 27, 39, and 52 in the non-tumor-forming group.

Supplementary Table S6. Differential proteins identified specifically on days 12 and 27 in the tumor-forming group (A) and the non-tumor-forming group (B).
